# Determination of CCT Diagram by Dilatometry Analysis of High-Strength Low-Alloy S960MC Steel

**DOI:** 10.3390/ma15134637

**Published:** 2022-07-01

**Authors:** Jaromír Moravec, Miloš Mičian, Miloslav Málek, Martin Švec

**Affiliations:** 1 Faculty of Mechanical Engineering, Technical University of Liberec, Studenstká 2, 461 17 Liberec, Czech Republic; jaromir.moravec@tul.cz (J.M.); martin.svec@tul.cz (M.Š.); 2 Faculty of Mechanical Engineering, University of Žilina, Univerzitná 8215/1, 010 26 Žilina, Slovakia; miloslav.malek@fstroj.uniza.sk

**Keywords:** high-strength steels, dilatometry analysis, CCT diagram

## Abstract

High-strength steels are used more than general structural steel due to their combination of properties such as high strength, good toughness and weldability. They are mainly used in the manufacture of heavy vehicles for the mining industry, cranes, transportation, etc. However, welding these grades of steel brings new challenges. Also, a simulation for welding high-strength steel is required more often. To insert a material database into the simulation program, it is necessary to conduct investigations using CCT (Continuous Cooling Transformation) diagrams, welded joints research, and more. To investigate the behavior of S960MC steel during heating and cooling, we used dilatometry analysis supported by EBSD (Electron Backscatter Diffraction) analysis. A CCT diagram was constructed. The transformation temperatures of A_c1_ and A_c3_ increase with increasing heating rate. The A_c1_ temperature increased by 54 °C and the A_c3_ temperatures by 24 °C as the heating rate increased from 0.1 °C/s to 250 °C/s. The austenite decomposition temperatures have a decreasing trend in the cooling phase with increasing cooling rate. As the cooling rate changes from 0.03 °C/s to 100 °C/s, the initial transformation temperature drops from 813 °C to 465 °C. An increase in the cooling rate means a higher proportion of bainite and martensite. At the same time, the hardness increases from 119 HV10 to 362 HV10.

## 1. Introduction

In previous decades, there was a considerable demand for lightweight but high-strength materials that led into the development of ultra-high-strength steels (UHSS), which belong to high-strength low-alloy (HSLA) steels. HSLA steels have excellent combination properties such as toughness, weldability, and a high strength–weight ratio. These steels enable usability in a wide range of industries including transportation vehicles, lifting devices (mobile cranes, lifting platforms), the oil and gas industry, automotive, shipbuilding, and shore constructions. The main advantage of HSLA steel application is reducing construction weight, e.g., a weight reduction of 60% is achieved by replacing conventional construction steel S355 with HSLA steel of grade S960MC [1,2,3,4,5]. HSLA steel consists of ferritic-perlite, ferrite-bainite, bainite, or martensitic (eventually annealed martensite) structure, thanks to which the desired properties are achieved. The industrial use of these steels is increasing, leading to reduced costs while maintaining all the required properties of the steels. HSLA steel characteristics include a combination of high strength and high toughness, good resistance against cold cracks, cold formability, and low carbon equivalent values [6]. HSLA steels can be classified as microalloyed steels because they also contain a small amount of alloying elements, e.g., aluminum (Al), titanium (Ti), niobium (Nb), and vanadium (V) [7]. Microstructures depend on the state of austenite before transformation, chemical composition (not only alloying elements), and cooling conditions. Various mechanical properties of steels are achieved by different combinations of microstructures and their mutual ratio [1]. Austenite can transform into all the aforementioned microstructures in microalloyed steels depending on the chemical composition and the cooling rate. The final microstructure is affected by the grain size of austenite before the transformation. Refined austenitic grains in microalloyed steels are critical in achieving a fine grain microstructure with high strength and toughness. Small precipitates effectively reduce the growth of austenitic grains during steel heating. The more stable the precipitation, the more grain growth is suppressed at higher temperatures [8].

The typical carbon content of these steels ranges from 0.05 to 0.25%, less than 2% of manganese, and a small amount of other elements such as chromium, nickel, molybdenum, copper, nitrogen, vanadium, niobium, titanium, and zirconium [7,9]. The maximum combined content of the elements titanium, vanadium, and niobium was up to 0.22% according to EN 10149-2. In particular, Ti, V, and Nb, together with carbon and nitrogen, form nitrides, carbides, and carbonitrides. The influence of each particle strongly depends on their stability and solubility at a certain temperature while processing steel [9]. The alloying element Nb is used for grain refinement and precipitation hardening. The particles NbC and Nb(C,N) are precipitated during hot forming [10,11]. Titanium reacts with nitrogen during the first process of melting steel. A small amount of Ti causes a fine dispersion of Ti nitride nanoparticles that prevent the growth of austenitic grains at high temperatures, 1200 °C [10,12]. Vanadium is more soluble in austenite than niobium. It precipitates in clumps in ferrite, forming fine precipitates, which harden the steel. Grain refinement is required; otherwise, the toughness of the steel reduces due to hardening [13].

Thermomechanical controlled processing (TMCP) is a technological process of controlled rolling at transformation temperatures from austenite to bainite or martensite. TMCP can be split into two steps: controlled rolling and controlled cooling [14]. The first rolling process takes place in the austenitic region at high temperatures. The material is still in the temperature range where recrystallization occurs. The second rolling of the final material thickness starts at temperatures where the recrystallization process does not occur and ends in the intercritical region (γ+α phase). The rolling process in the intercritical region causes grain deformation (ledges) and deformation bands. Deformed austenite does not regenerate and keeps the high dislocation density. During the transformation, the formed deformation ledges and bands act as nucleation sites and lead to grain refinement. The accelerated controlled cooling follows after the final rolling (at the intercritical region) [12].

The combination of a controlled rolling process and low alloying element content (including carbon content) provides these steels with good weldability while maintaining the low construction weight and high joint strength [12,14]. However, the welding of TMCP steels is more demanding than other common structural steels because stricter technological parameters are required. Due to welding, microstructural changes occur in areas affected by the thermal effect during the welding process. This heat-affected zone (HAZ) significantly influences welded joints; therefore, monitoring and evaluating HAZ is one way to control the quality of welded joints. Heat input, cooling rate (time t_8/5_), and the type of additional material are critical welding parameters affecting the final properties of HAZ. The heat-affected zone can be divided into four base regions: coarse-grained heat-affected zone (CGHAZ), fine grains heat-affected zone (FGHAZ), intercritical heat-affected zone (ICHAZ), and sub-critical heat-affected zone (SCHAZ).

A typical sign of HAZ-welded joints manufactured from HSLA steels is the presence of a soft zone. There is a soft transformation zone when the peak temperature exceeds A_C1_ and a tempering soft zone when the temperature does not exceed A_C1_. These soft zones are typical for high-strength steels, with a lower hardness than the base material and a reduction in yield strength [15,16,17,18,19].

Nevertheless, this steel is possible to weld with suitable welding methods such as laser or electron beam welding, hybrid welding technologies, or modern advanced arc welding technologies [6,20,21,22].

Gas metal arc welding (GMAW) is one of the most used welding techniques. The conventional GMAW methods decrease the HAZ’s mechanical properties against the base material. High heat input causes a slower cooling rate and decreases material hardness at HAZ [3,23].

For this research paper [20], we compared the welding of S960MC steel with an 8 mm thickness using different methods (EB+CW—electron beam welding with cold wire, LH—laser hybrid welding, P+CW—plasma arc welding with cold wire, and GMAW). The most significant decreasing value of the hardness was observed in the heat-affected zone, especially in the ICHAZ region. The goal of the mentioned investigation was to use the non-match additional material and reach suitable welding joints achieved by laser and electron beam welding. The next investigation [23,24,25,26] involved laser welding of S960MC steel with 8 mm thickness for one pass. A significant drop in hardness was detected in the SCHAZ region, which was very narrow. The hardness value measured in other sub-regions of HAZ was higher than the base material. Nonetheless, the sample broke at the base material during the tensile strength test, far enough from HAZ. The authors considered that the weld created by the laser beam was narrow enough to achieve the behavior of all HAZ subzones as one heat-affected zone. This phenomenon was observed and confirmed by another investigation [2,13,16,18,19]. The effects of different heat inputs on the welding joints were investigated in a research paper [27]. GMAW technologies were applied to create S960MC steel joints with a 3 mm thickness. The most critical sub-zones of HAZ are ICHAZ and SCHAZ. The width of HAZ and the toughness of the welded joint increased with higher heat input simultaneously.

Phase transformation diagrams are created for specific steels to record austenite’s transformational changes during the cooling process in the temperature and time axes (eventually log time). The phase transformation diagrams depend on the chemical composition of the steel. These diagrams are applied in the fields of heat treatment, manufacturing, and the development of steel or welding [28]. The construction of a CCT diagram can be achieved in several ways. In the research papers [29,30,31,32,33,34,35,36], a CCT diagram was constructed using dilatometric measurements, structural analysis, and hardness measurements. In certain cases, in technical practice, the prediction of the CCT diagram is also sufficient, especially for expensive materials, or lack of time. Another way to obtain a CCT diagram is to use a software program, as was the case in the article [33], where the results were compared with commercial software JMatPro and experimentally determined by CCT diagram. In the investigation [4] article, the same S960MC steel was examined, but with a thickness of 8 mm, to determine the decay structures due to different cooling rates. An ARA diagram was constructed using dilatometric analysis, microstructural analysis, and hardness measurement.

Based on the results obtained in the research [4,29,30,31,32,33], dilatometric analysis, together with hardness measurement and metallographic analysis, was selected as the most suitable method for accurate determination of the CCT diagram.

## 2. Materials and Methods

### 2.1. Experimental Material

The structural steel S960MC, manufactured by SSAB, was the material we chose to investigate. It is a thermo-mechanically controlled processed (TMCP) steel designed for cold forming at 400 °C [34]. The technical parameters, chemical composition, and delivery conditions should fulfill the requirements of the STN EN ISO 10149-2:2014 standard [37]. The first measurement executed was a spectral analysis to detect the chemical composition (average value from three measurements) of investigated steel shown in Table 1 compared to the STN EN ISO 10149-2:2014 standard and material certificate. The mechanical properties (elongation, yield, and tensile strength) were also measured and compared to the STN EN ISO 10149-2:2014 standard in Table 2. Samples for tensile strength tests were prepared from the steel sheet in three directions based on the rolling process: longitudinally (0°), diagonally (45°), and transversely (90°) in the rolling direction. Three samples were taken for each orientation. The samples for the mechanical properties test were prepared and tested according to the STN EN ISO 6892-1:2022 with an INSTRON Series 5985 at ambient temperature 22 °C [38]. The maximum force of this equipment at full speed is 125 kN, force measurement accuracy is ± 0.4% of reading down to 1/100 of load cell capacity and displacement measurement accuracy is ± 0.01 mm of displacement. The values of the anisotropy coefficient showed that the base material did not show any signs of planar anisotropy in all investigated parameters (tensile strength—R_m_, yield strength—R_p0.2_, and percentage elongation after fracture—A).

**Figure 1:**
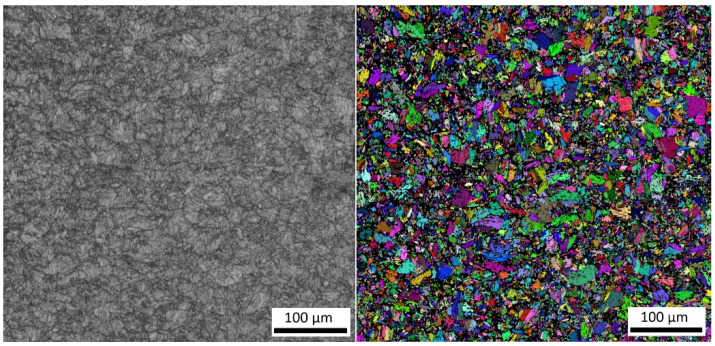
The martenzite–bainitic structure (**left**) and EBSD grain size analysis (**right**) of tested S960MC steel (HV 20 kV, step size 0.2 um).

The S960MC steel is a microalloyed, thermo-mechanically processed, high-strength structural steel with a microstructure consisting of martensite, bainite, and their tempered variants. Figure 1 (left) shows the microstructure of experimental steel; Figure 1 (right) shows the electron backscatter diffraction (EBSD) grain size analysis. The resulting mean grain size of the supplied material was 4.1 µm with standard deviation 2.3 µm. This kind of microstructure provides a good combination of high tensile strength and fracture toughness.

### 2.2. Preparation of Samples for Dilatometric Analysis

The dilatometry tests were conducted with a Quenching Dilatometer DIL 805L at the Technical University of Liberec. It is fully automated, containing self-contained units used to measure dimensional changes under extreme controlled heating and cooling conditions. The inductive heating with constant sinus frequency is power controlled by amplitude adjustment for superior temperature homogeneity in the sample. The heating sample, ranging from 20 °C up to 1500 °C, can operate under different environmental conditions of the chamber, such as air, vacuum, or special inert gases (especially argon, helium, and nitrogen). The minimum possible temperature is −160 °C in specific boundary conditions. The highest cooling rate is achievable by helium as the cooling medium and high heat conductivity [39]. Resolution when measuring length change is ΔL/°C = 0.01 µm/0.05 °C.

**Figure 2:**
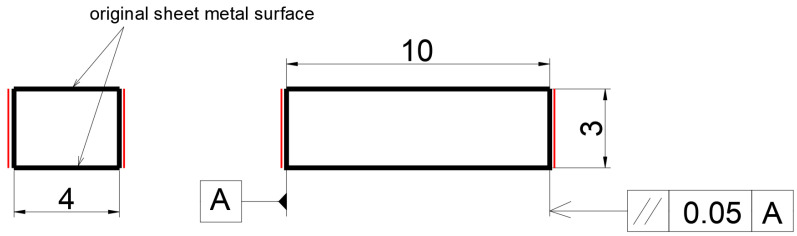
The schematic shape of samples for the dilatometric test with red lines indicating the machined sides.

The samples for the dilatometry test were prepared from the sheet metal longitudinal to the rolling direction. In this case, we used prism-shaped samples with a rectangular base, Figure 2. Flat-shaped samples for the dilatometry test are used for thin materials, usually with a thickness of >4 mm [6,35,36]. Opposite surfaces (after a longer edge) of the test sample must be parallel and surface roughness of 5 µm or less is required. These surfaces were made by milling, followed by grinding (red line indicators). The investigated steel had a 3 mm thickness, and the sides were not machined.

### 2.3. Determination of the Dilatometric Curve

For the dilatometric tests, 13 variants of different combinations for heating rate, delay at the austenitizing temperature, and cooling rate were used. They all shared an austenitization temperature of 1100 °C. The austenitization temperature delay was set to 30 s (only two variants were performed without delay). The heating for the dilatometric tests was controlled by a programmed temperature with a control step of 0.25 °C, i.e., four values are recorded when the programmed temperature increases by 1 °C. A control step of 0.05 °C was set for the cooling part of the dilatometric curve to obtain a more detailed analysis of austenite decomposition. Thus, 20 values were recorded when the programmed temperature increased by 1 °C. Two samples represented each variant. The heating and cooling rate parameters and all variants are listed in Table 3 (aligning with increased cooling rate).

**Figure 3:**
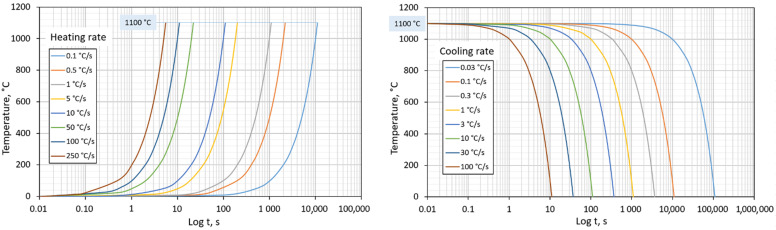
Different program temperature cycles for the control dilatometer DIL 805L; **left**—heating phase; **right**—cooling phase.

We examined the following effects via dilatometry analysis:

1. The heating rate’s effect on shifts in the transformation temperatures, A_c1_ and A_c3_. For this experiment, eight variants with different heating rate values were useds from 0.1 °C/s to 250 °C/s. The cooling method was not considered. These variants are listed in Table 4 (aligning with increasing heating rate). Program control cycles are shown in Figure 3 (left).
2. The cooling rate affects the resulting austenite transformation temperatures in the cooling phase, microstructure, and hardness. We used eight variants with different cooling rates from 0.03 °C/s to 100 °C/s for this experiment. The heating method was not considered. These variants are listed in Table 5 (aligning with increasing cooling rate). Program control cycles are shown in Figure 3 (right). Subsequently, a CCT diagram was created from the data analysis.
3. The effect of heating and cooling rates on the resulting grain size. All variants listed in Table 3 were used for this experiment. Out of these, variants have a constant heating rate but a different cooling rate. Variants with significantly different heating rates were used but with a constant cooling rate. We assessed which parameter (heating or cooling rate) would have a greater effect on the resulting grain size.

The following descriptive statistics were used to process the experimental data: average value and standard deviation (as a measure of variability) if the measured quantity was measured multiple times. Next, regression analysis was used. The aim of this analysis is to clarify the relationship between the input variable (heating rate or cooling rate) and the output measured variables (temperatures A_c1_ and A_c3_, transformation temperatures of austenite and grain size). The influence of the input parameters must be verified at low levels of values, as well as at high ones. For this reason, the input parameters were chosen in a logarithmic series. A logarithmic function was used as a mathematical model to describe functional dependence. Furthermore, the Shapiro-Wilk test was used to confirm the normality of the regression residues.

### 2.4. Methodology for Determining Phase Transition Temperatures from Dilatometry Curves

The phase transformations were evaluated from the heating and cooling parts of the dilatometry curves and recorded in the dilatation and temperature axes. Non-linear changes to the length indicated phase transformation. For determining transformation temperatures, the three tangent method and the first derivative method were used. Both methods were used in several other studies and show good agreement of results [36,40,41,42,43].

The three tangent methods can also identify multiple phase transformations and is suitable for determining transition temperatures of austenite decomposition. The first derivation of the dilatometry curve method was used as the second method for evaluating the phase transition temperatures. In this case, the phase change temperature was determined when the function of the first derivative of the dilatometry curve acquired a value of zero.

### 2.5. Microstructure Observation and Hardness Measuring

The dilatometric samples were cut in half, perpendicular to the long side of the sample, for the metalographic analysis and hardness test. Samples prepared in this way were embedded in PolyFast resin with additional carbon content to secure electrical conductivity for the electron microscope. Samples were prepared with the SimpliMet 1000 using the following process parameters: heating temperature 180 °C, exposure time 4.5 min, cooling time 3.5 min, and 250 bar pressure. Samples for the metallographic analysis were prepared on grinding paper with grid FEPA P240, P500, P800, P1200, P2000, and P4000. Polishing was conducted by diamond suspension with average grain sizes of 3 and 1 µm. The oxide polishing with OP-S suspension and 0.25 µm were used for the final polishing. The samples’ structural analysis was performed on an optical microscope Olympus DSX 500. Photos of the microstructure were processed by NIS—Elements software.

The grain size measurement was determined by the electron microscope Tescan SEM Mira 3, which was equipped with a local chemical analysis detector (EDX) Oxford UltimMax65 and EBSD detector Oxford Symmetry. Detector with its software-controlled tilting, can be positioned at the ideal geometry for every sample type, from TEM foils to cm—scale samples and can satisfy these criteria—full 1244 × 1024-pixel diffraction patterns are ideal for high angular resolution (HR) EBSD. Measurements were performed using the following process parameters: accelerating voltage of 20 kV and a measured area of 500 × 500 µm with a measuring step of 0.2 µm. The equivalent circle diameter (ECD) parameter was used for grain size analysis, which is determined by the complete grain area.

Similar to the microstructure analysis, hardness was measured for the surfaces of the test samples. The Vickers method’s hardness measurement involving a load of 98.07 N (HV10) was executed in the laboratory using a fully automatic micro-hardness tester Qness Q30A with a load capacity from 0.98 N up to 306.6 N (HV0.1 up to HV30). Test force tolerance is <0.5% for all test forces. The HV10 hardness measurement was performed on cross-sections (the dilatometric sample was cut in two halves on the long side) at ambient temperature 22 °C. Hardness was measured on the two surfaces, with 3 measurements on one surface and 3 on the other, a total of 6 measurements for one variant.

## 3. Results and Discussion

### 3.1. The Heating Rate’s Effect on Shifts in the Transformation Temperatures A_c1_ and A_c3_

The austenite transformation temperature A_c1_ and full austenitization temperature A_c3_ were determined from eight dilatometry curves. We used both methods for determining temperature—the three tangent method and the first derivation of the dilatometry curve method.

**Figure 4:**
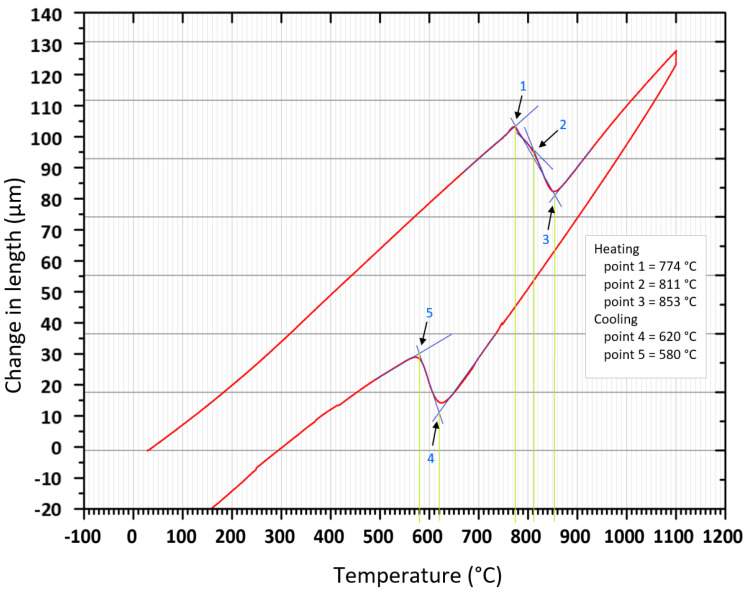
Dilatometry curves for 10 °C/s heating rate and 1 °C/s cooling rate (variant H10 C1) with three tangents applied.

Figure 4 shows the whole dilatometry curve for a heating rate of 10 °C/s and a cooling rate of 1 °C/s (variant H10 C1). Points 1–3 are the temperatures in the heating phase when the base material’s original structure austenitizes. Two temperatures (points 4 and 5) are determined in the cooling phase representing the phase transformation during austenite decomposition.

**Figure 5:**
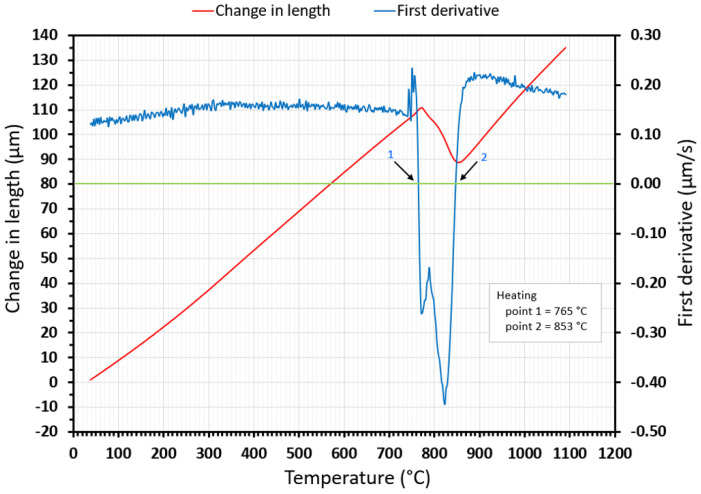
The heating part of the dilatometry curve for a 10 °C/s heating rate with applied first derivative.

Figure 5 shows the heating part of the dilatometry curve (red curve) when heated at a rate of 10 °C/s. In the same graph, the first derivative is shown in blue. Points 1 and 2 are the transformation temperatures (A_c1_ and A_c3_) at which the first derivative acquires zero.

**Figure 6:**
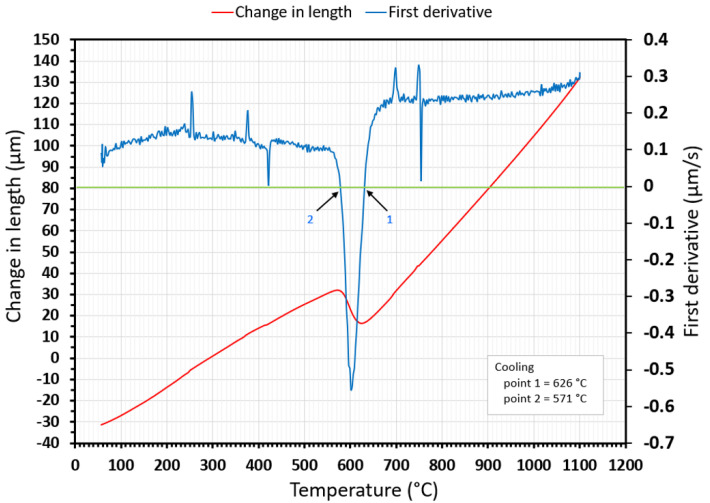
The cooling part of the dilatometry curve for a 1 °C/s cooling rate with the first derivative applied.

Figure 6 shows the cooling part of the dilatometry curve during cooling at a rate of 1 °C/s (red curve) and the first derivative of this section for the dilatometry curve (blue curve). Points 1 and 2 are the transformation temperatures of austenite decay when the first acquires the value zero. The transformation’s beginning and end indicate the alloy phase boundaries, e.g., ferrite, carbide, pearlite, bainite, martensite, or other eutectoid phase batches. We used the same procedures to evaluate all dilatometric curves.

**Figure 7:**
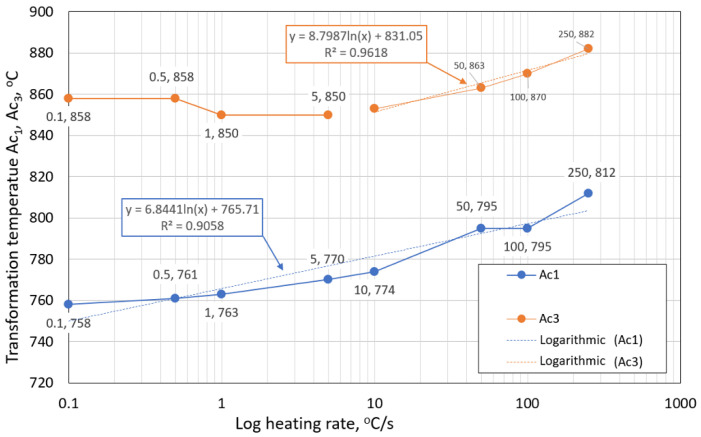
The influence of the heating rate on temperatures A_c1_ and A_c3_.

The deviation of the first derivation method from the tangent method was a maximum of 2.1%. Values from the three tangent method were used in further analyses. Table 6 lists all analyzed temperatures. Figure 7 shows the influence of the heating rate on temperatures A_c1_ and A_c3_.

**Figure 8:**
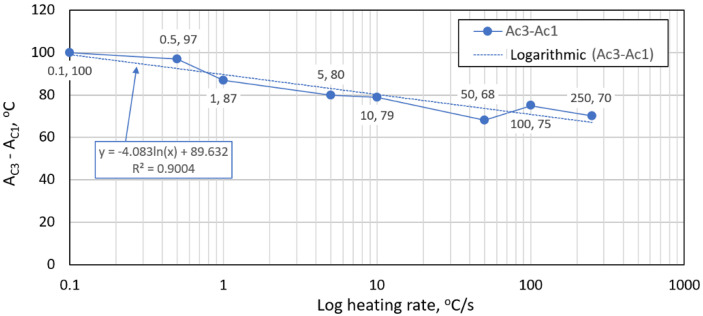
The influence of the heating rate on temperature differences between A_c3_ and A_c1_.

The austenitic transformation (A_c1_ temperature) increases with an increased heating rate. This trend was observed in other works [36,43]. The A_c3_ temperature, when complete austenitization is achieved, was almost constant up to a heating rate of 10 °C/s. At heating rates higher than 10 °C/s, it began to grow. The differences between A_c3_ and A_c1_ decreased with an increased heating rate, as shown in Figure 8. The phenomena described are important in creating the width of the heat-affected zone and welding process modeling.

### 3.2. Austenite Transformation Temperatures in Cooling Phase

Determining the transformation temperatures of austenite decomposition was crucial for the later design of the S960MC steel CCT diagram. Eight cooling dilatometry curves were used. The deviation of the first derivation method from the tangent method was a maximum of 3.7%. Other transformation temperatures, T_2_ and T_3_, occurred in addition to the first -T_1_ and last -T_4_ transformation temperatures and were also evaluated from the three tangent method. Curves with multiple transformation temperatures and optical microstructural analyses were important in determining all the excluded structural phases. The transformation temperatures are provided in Table 7.

**Figure 9:**
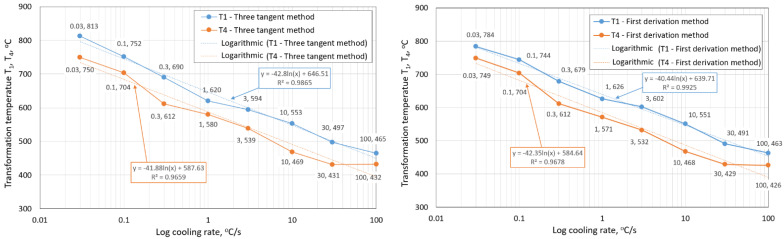
The influence of the cooling rate on transformation temperatures T_1_ and T_4_: **left**—three tangent method; **right**—first derivation method.

During slow cooling rates, austenite decomposition occurred at high temperatures, slightly under the A_C1_ temperature. A slow cooling rate and high transformation temperature are typical signs of diffusion transformation of austenite to ferrite or perlite, particularly in the case of low-carbon and low-alloyed steel [4,6,36,44,45,46]. Austenite decomposition occurs at a lower temperature with an increasing cooling rate, indicating a bainite or martensite structure. This process occurred with the shear transformation of austenite [44]. Hardness measurements for all samples were performed to compare with the microstructure while considering hardness the ferrite–perlite steel structure, next bainite, martensite and their mixture [4,6,36,47]. The cooling rate’s effect on the values of the first and last austenite decomposition transformation temperature for both methods of determination is shown in Figure 9.

### 3.3. The Analysis of the Hardness after Cooling

**Figure 10:**
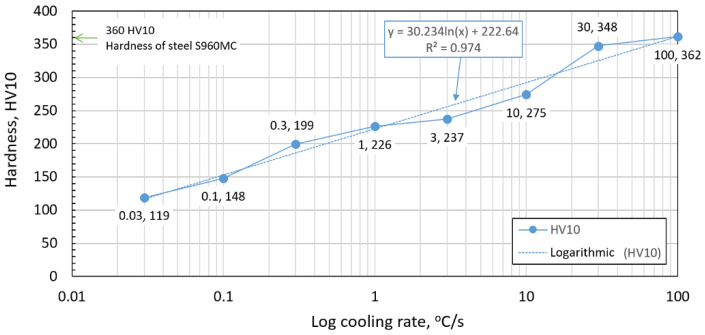
The influence of the cooling rate on material hardness.

The hardness of the final microstructure for different cooling rates is recorded in Table 8. The sample’s average hardness value for a 100 °C/s cooling rate corresponded with the hardness of the base material. The base material’s microstructure is predominantly martensitic and was the same for the 100 °C/s cooling rate, the typical cooling rate for hardening HSLA steels [36,43,45,47,48]. The first significant hardness drop was recorded at a 10 °C/s cooling rate with a 23% decrease compared with the base material. As the cooling rate decreased, the hardness of the microstructure also decreased. Figure 10 shows the influence of the cooling rate on material hardness. The approximate hardness value of the base material is reached at a cooling rate between 30 and 100 °C/s. A rising cooling rate did not increase hardness; therefore, a cooling rate of 100 °C/s was set as the critical cooling rate. On the contrary, decreasing the cooling rate under 0.03 °C/s did not significantly decrease material hardness.

### 3.4. The Microstructural Analysis and Design of CCT Diagram

**Figure 11:**
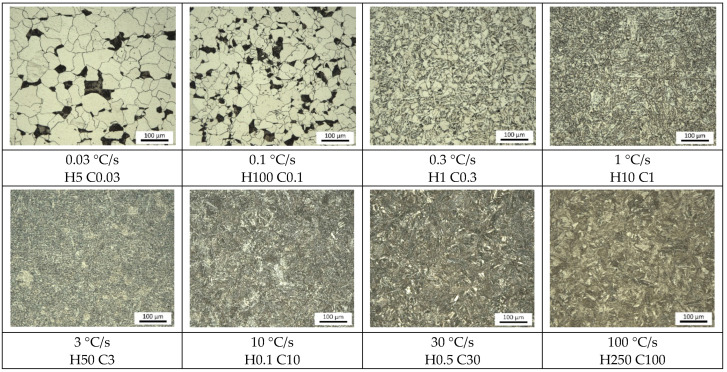
The influence of the cooling rate on the microstructure of S960MC steel.

A microstructural analysis confirmed the occurrence of phases such as martensite and bainite and their temperate variants, perlite or ferrite. Microstructures at different cooling rates are shown in Figure 11. A slow cooling rate (0.03 °C/s, 0.1 °C/s, and 0.3 °C/s) led to an average grain size growth, and the structure consisted of ferrite and perlite. The proportion of perlite increased with the increased cooling rate; it had a structure of about 60% at the cooling rate of 0.3 °C/s. We performed this analysis using NIS-Elements software.

The microstructure of steel at a cooling rate of 1 and 3 °C/s comprises a mixture of ferrite and bainite (bainite predominates). Martensite appears in the microstructure only at a cooling rate of 10 °C/s. Its content increases with the increased cooling rate. At a cooling rate of 30 °C/s, its content is at the level of 90%.

**Figure 12:**
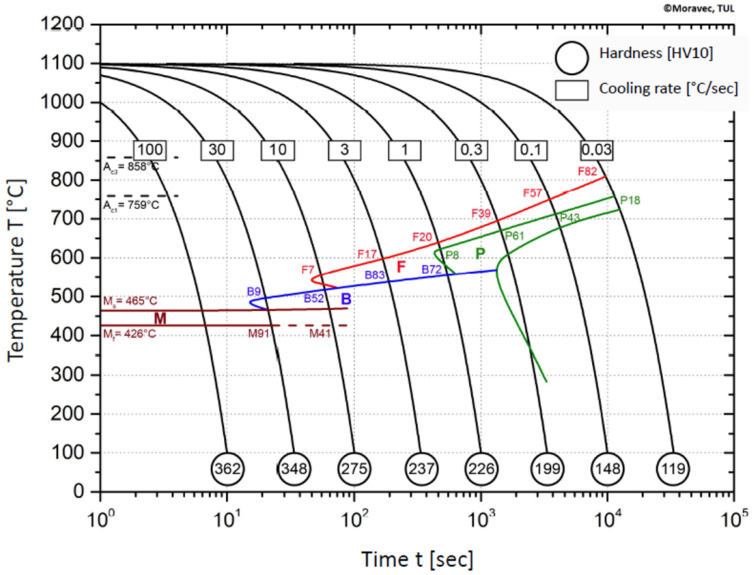
The CCT diagram of S960MC steel.

Based on the transformation temperatures of austenite decomposition and microstructure (with hardness values), it was possible to design a CCT datagram for the structural steel S960MC. The resulting diagram is shown in Figure 12. It should be noted that the diagram is valid for a range of chemical compositions provided in Table 1. Furthermore, it only applies to previous mechanical properties shown in Table 2, which reflect the processing method during production.

A similar diagram for S960MC steel is provided in [4]; however, differences in chemical composition do not correspond to the diagram shown in Figure 12.

### 3.5. The Effect of Heating and Cooling Rates on the Resulting Grain Size

**Figure 13:**
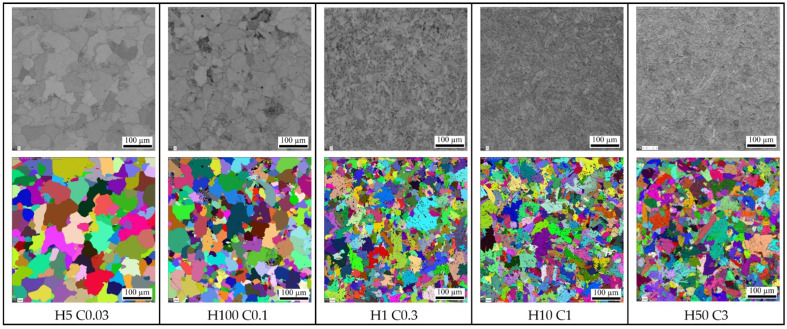

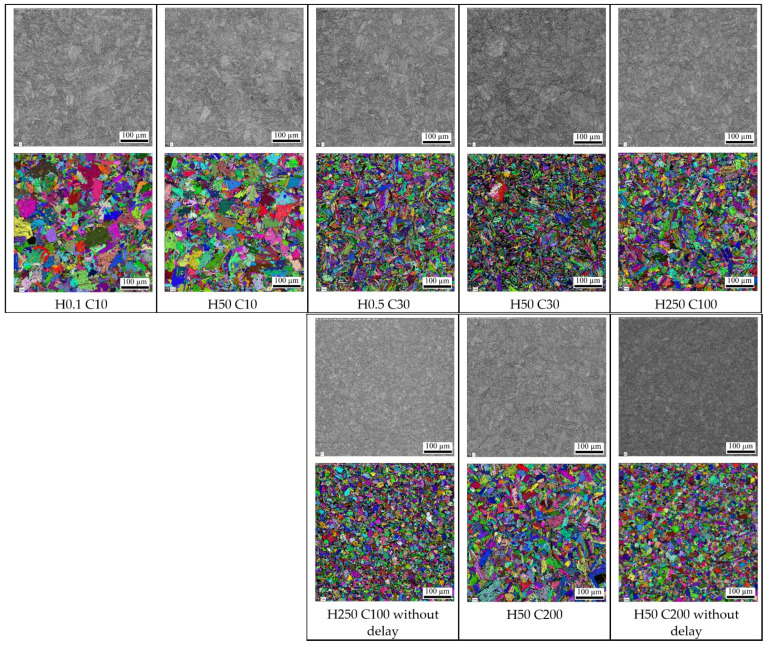
SEM micrographs and EBSD analysis of grain size at different variants of the heating rate and cooling rates.

**Figure 14:**
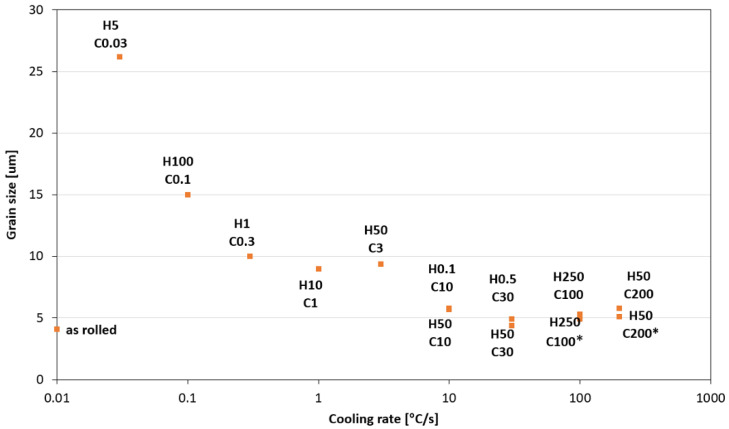
The EBSD analysis of grain size for different heating and cooling rates.

The mechanical properties of high-strength structural steel correspond to their grain size (e.g., via the Hall–Petch relationship); strength is inversely dependent on the square root of grain size. Although conventional methods for determining grain size using optical microscopy are well-established, EBSD methods offer many advantages over these techniques, including increased spatial resolution and a quantitative description of the grains’ orientation. Figure 13 is a comprehensive overview of the analyzed SEM microstructures and the EBSD grain size analysis results for all thirteen heating and cooling rate combinations. A band contrast filter was used for the SEM’s microstructural analysis, which is the base for EBSD. Table 9 shows the grain size values after EBSD analysis for the equivalent circle diameter (ECD). The values from Table 9 are graphically presented in the graph in Figure 14.

Considering these measured values, we can state that the cooling rate significantly affects grain size. Variants (H50 C3, H50 C10 and H50 C30) confirmed this finding when the heating rate was constant, equal 50 °C/s, and grain size decreased from 9.4 µm to 4.4 µm with the rising cooling rate (3 °C/s, 10 °C/s and 30 °C/s). This means that when the heating rate is increased by 10 times, the grain size is reduced by 53%. It can also be stated that the heating rate is not significant for grain growth. The cooling rate was constant, equal 10 °C/s, for variants H50 C10 and H0.1 C10, and the heating rates were 0.1 and 50 °C/s at the grain size (5.7 µm and 5.8 µm). Despite the high change in heating rate, the grain size change was at the level of 2%. It was also confirmed at other tests for the constant cooling rate of 30 °C/s and heating rates of 0.5 and 50 °C/s. The grain size was 4.4 µm and 4.9 µm, which is the difference <10%.

The effect of delay on the austenitization temperature of 1100 °C was investigated on pairs of variants H250 C100 and H50 C200. One of the pair of variants was allowed to stand at the austenitization temperature for 30 s. The difference in grain size was 8% for the H250 100 variant and 14% for the H50 C200 variant. It can be stated that this effect is much smaller than the effect of the cooling rate. A more pronounced effect of delay at the austenitization temperature on grain growth would be manifested at higher time values from 30 min and more. However, these values are not applicable to the welding area.

Dramatic changes in the grain size were observable until the 30 °C/s cooling rate closest to the base material. The measured grain size of the base material was 4.1 µm. Further increases in cooling rate did not result in a reduction in grain size. At a speed of 30 °C/s, the structure was already 91% martensite and the higher speed no longer brought significant changes in structure and hardness.

## 4. Conclusions

Our research paper focused on determining transformation temperatures by dilatometry analysis, the effect of the cooling rate on hardness, grain size, and creation of the final microstructure. Furthermore, the heating rate’s effect on shifts in the transformation temperatures A_c1_ and A_c3_ was investigated. The influence of the cooling rate on the final microstructure properties was also investigated. The executed measurements and experiments led to the creation of a CCT diagram for structural steel S960MC with a 3 mm thickness and specific chemical composition stated in Table 1. The main conclusions of the above research are as follows:

- Both methods, the three tangent method and the first derivation of the dilatometry curve method, are suitable for determining transformation temperatures. The deviation of the first derivation method from the tangent method was a maximum of 2.1% (determination of transformation temperatures in the heating phase) and a maximum of 3.7% (determination of transformation temperatures in the cooling phase).
- The austenitic transformation (A_c1_ temperature) increases with an increased heating rate. The dependence of these two parameters was tested by regression analysis. A linear regression model with a logarithmic function was used, which demonstrated the strong dependence of parameters (coefficient of determination was R^2^ = 0.9). This analysis shows that in the range of heating rates from 0.1 to 250 °C/s, the temperature A_c1_ increased from 749 °C to 804 °C.
- The A_c3_ temperature, when complete austenitization is achieved, was almost constant up to a heating rate of 5 °C/s (average value was 854 °C and standard deviation was 4 °C). At heating rates higher than 10 °C/s, it began to grow. From a heating rate of 10 °C/s to 250 °C/s the dependence was also described by a logarithmic function with coefficients of determination equal to R^2^ = 0.96. The phenomena described are important in creating the width of the heat-affected zone and welding process modeling. The welding processes with a high heating rate of the base material (laser welding and electron beam welding) will be characterized by a narrow HAZ. This eliminates the “softening effect of HAZ” that is typical for HSLA steel welding.
- The dependence of the cooling rate on the transformation temperatures T1 and T4 of austenite decomposition is significant and has the opposite trend as in the heating phase. As the cooling rate increases, the austenite decomposition temperature decreases. A linear regression model with a logarithmic function was used, which demonstrated the strong dependence of parameters (coefficient of determination was R^2^ = 0.98 for temperature T1 and R^2^ = 0.97 for temperature T4). The data are valid for the three tangent method. This analysis shows that in the range of cooling rates from 0.03 °C/s to 100 °C/s, the temperature T1 increased from 796 °C to 449 °C and temperature T4 increased from 734 °C to 394 °C.
- A slow cooling rate and high transformation temperature are typical signs of diffusion transformation of austenite to ferrite or perlite. The microstructure of steel at a cooling rate of 1 °C/s and 3 °C/s comprises a mixture of ferrite and bainite (bainite predominates). Martensite appears in the microstructure only at a cooling rate of 10 °C/s. Its content increases with the increased cooling rate. At a cooling rate of 30 °C/s, its content is at the level of 91% and at a cooling rate of 100 °C/s the microstructure is fully martensitic.
- The hardness of the samples increased with the increased cooling rate due to the higher percentage of hardness phases such as bainite and martensite. The dependence of these two parameters was tested by regression analysis as well. A linear regression model with a logarithmic function was used. The coefficient of determination was R^2^ = 0.97, which means a strong tightness of the variables and a well-chosen regression function. This analysis shows that in the range of cooling rates from 0.03 °C/s to 100 °C/s, the hardness increased from 116 HV10 to 362 HV10. The hardness of the base material 360HV10 will be achieved according to this model at a cooling rate of 94 °C/s. This cooling rate represents a time t_8/5_ of 3.6 s, which can be achieved by using concentrated heat source welding methods.
- As the cooling rate increases, a trend of decreasing average grain size can be observed. However, regression analysis did not show strong parameter tightness using the full cooling rate range (from 0.03 °C/s to 100 °C/s). The coefficient of determination was R^2^ = 0.72 for the logarithmic regression function and R^2^ = 0.85 for the power regression function. Data selection and analysis, however, demonstrated these findings. We can state that the cooling rate significantly affects grain size. Variants (H50 C3, H50 C10 and H50 C30) confirmed this finding when the heating rate was constant (50 °C/s), and grain size significantly decreased from 9.4 µm to 4.4 µm with the rising cooling rate (from 3 °C/s to 30 °C/s). This represents a decrease of 53% with a cooling rate change of 27 °C/s. The effect of the heating rate is not significant on the change in grain size. The cooling rate was constant for variants H50 C10 and H0.1 C10 (10 °C/s), and the heating rates were 0.1 and 50 °C/s at the same grain size (5.7 µm and 5.8 µm). The difference in grain size in these two variants is up to 2% with a change in heating rate of approximately 50 °C/s. It was also confirmed at the tests for the cooling rate of 30 °C/s and heating rates of 0.5 and 50 °C/s. The difference in grain size for both cases was <10%.
- The data presented in the graphs in Figure 7, Figure 8, Figure 9 and Figure 10 were subjected to the Shapiro-Wilk test. In all cases, the normality of the residue distribution was confirmed.
- The CCT diagram is valid for the range of chemical compositions provided in Table 1. It can only be applied to the mechanical properties recorded in Table 2, reflecting the processing method during production.

## Figures and Tables

**Table 1:** Chemical composition of investigated steel.

According	Chemical Composition wt.%—Strenx 960MC										
	C	Si	Mn	P	S	Al	Nb	V	Ti	Mo	B
EN 10149-2 *	0.200	0.60	2.200	0.025	0.010	0.015	0.090	0.200	0.250	1.000	0.005
Material certificate **	0.085	0.180	1.060	0.010	0.003	0.036	0.002	0.007	0.026	0.109	0.001
Experimental measurement average value/standard deviation	0.055/ 0.0024	0.168/ 0.0045	1.203/ 0.0047	<0.010	<0.010	0.037/ 0.00023	<0.005	0.005/ 0.00025	0.023/ 0.00017	0.086/ 0.00012	-
	Cu	Cr	Ni	N	CEV	CET					
EN 10149-2 *	-	-	-	-	-	-					
Material certificate **	0.010	1.080	0.070	0.005	0.506	0.258					
Experimental measurement average value/standard deviation	<0.005	1.056/ 0.0081	0.046/ 0.0017	-	0.489	0.238					

* Maximum amount of alloying elements except Al. The prescribed Al content is its minimum amount. The sum of the elements Nb, V, and Ti must not exceed 0.22%. ** According to EN 10204-3.1 inspection certificate supplied by the manufacturer.

**Table 2:** Mechanical properties of S960MC steel.

According	Angle of Rolling Direction	Mechanical Properties S960MC, Thickness 3 mm						
		R_p0.2_ [MPa]	R_m_ [MPa]	R_p0.2_/R_m_	A [%]			
		
EN 10149-2	-	min. 960	980–1250	-	min. 7			
		Average R_p0.2_ [Mpa]/Standard deviation [Mpa]	Average R_m_ [Mpa]/Standard deviation [Mpa]	Average R_p0.2_/Average R_m_	Average A [%]/Standard deviation [%]	Coefficient of area anisotropy P * [%]		
						PR_m_	PR_p0.2_	PA
Experimental measurement	0°	1007/15.6	1092/7.3	0.92	7.9/0.28	-	-	-
	45°	1018/5.1	1106/7.4	0.92	6.7/0.16	1.2	1.1	−14.4
	90°	1044/7.3	1124/4.6	0.93	6.5/0.05	2.9	3.6	−17.0

* The area anisotropy coefficient was calculated from the average values of R_p0.2_, R_m_ and A.

**Table 3:** Heating and cooling rate variants in the dilatometric tests.

**Variant Designation**	**H5 C0.03**	**H100 C0.1**	**H1 C0.3**	**H10 C1**	**H50 C3**	**H0.1 C10**	**H50 C10**
Heating rate °C/s	5	100	1	10	50	0.1	50
Cooling rate °C/s	0.03	0.1	0.3	1	3	10	10
**Variant Designation**	**H0.5 C30**	**H50 C30**	**H250 C100**	H250 C100 *	**H50 C200**	H50 C200 *	
Heating rate °C/s	0.5	50	250	250 *	50	50 *	
Cooling rate °C/s	30	30	100	100 *	200	200 *	

* Sample without delay at austenitizing temperature 1100 °C.

**Table 4:** The chosen variants for analyzing the shift in transformation temperatures A_c1_ and A_c3_.

Heating Rate °C/s	0.1	0.5	1	5	10	50	100	250
Cooling rate °C/s	10	30	0.3	0.03	1	3	0.1	100
Variant designation	H0.1 C10	H0.5 C30	H1 C0.3	H5 C0.03	H10 C1	H50 C3	H100 C0.1	H250 C100

**Table 5:** The chosen variants for analyzing austenite transformation temperatures in the cooling phase, final microstructure, and hardness.

Cooling Rate °C/s	0.03	0.1	0.3	1	3	10	30	100
Heating rate °C/s	5	100	1	10	50	0.1	0.5	250
Variant	H5C 0.03	H100 C0.1	H1 C0.3	H10 C1	H50 C3	H0.1 C10	H0.5 C30	H250 C100

**Table 6:** The effect of the heating rate on shifts in the transformation temperatures A_c1_ and A_c3_.

Heating Rate [°C/s]		0.1	0.5	1	5	10	50	100	250
Variant Designation		H0.1 C10	H0.5 C30	H1 C0.3	H5 C0.03	H10 C1	H50 C3	H100 C0.1	H250 C100
Three tangent method	A_c1_ [°C]	758	761	763	770	774	795	795	812
	A_c3_ [°C]	858	858	850	850	853	863	870	882
First derivation method	A_c1_ [°C]	753	758	763	770	788	806	812	832
	A_c3_ [°C]	855	850	850	850	870	880	886	898
Difference between used methods	diff. A_c1_ [%]	0.66	0.40	0.00	0.00	1.78	1.36	2.09	2.40
	diff. A_c3_ [%]	0.35	0.94	0.00	0.00	1.95	1.93	1.81	1.78

**Table 7:** The transformation temperatures of austenite decomposition.

Cooling Rate [°C/s]		0.03	0.1	0.3	1	3	10	30	100
Variant Designation		H5C 0.03	H100 C0.1	H1 C0.3	H10 C1	H50 C3	H0.1 C10	H0.5 C30	H250 C100
Three tangent method	T_1_ [°C]	813	752	690	620	594	553	497	465
	T_2_ [°C]	-	744	-	-	-	534	465	456
	T_3_ [°C]	-	-	-	-	-	514	469	442
	T_4_ [°C]	750	704	612	580	539	469	431	432
First derivation method	T_1_ [°C]	784	744	679	626	602	551	491	463
	T_4_ [°C]	749	704	612	571	532	468	429	426
Difference between used methods	diff. T_1_ [%]	3.70	1.08	1.62	0.96	1.33	0.36	1.22	0.43
	diff. T_4_ [%]	0.13	0.00	0.00	1.58	1.32	0.21	0.47	1.41

**Table 8:** The hardness of the final microstructure for different cooling rates.

Cooling Rate [°C/s]	Variant	Hardness HV10						Average Hardness HV10/Standard Deviation HV10
0.03	H5C 0.03	118	116	122	117	119	123	119/2.54
0.1	H100 C0.1	152	145	157	144	148	143	148/4.94
0.3	H1 C0.3	196	201	203	197	200	199	199/2.36
1	H10 C1	223	231	227	227	227	223	226/2.75
3	H50 C3	236	238	240	232	237	239	237/2.58
10	H0.1 C10	276	268	279	281	273	271	275/4.50
30	H0.5 C30	349	344	352	352	346	342	348/3.81
100	H250 C100	357	365	356	372	361	360	362/5.40
Base material		362	363	357	362	360	358	360/2.21

**Table 9:** The chosen variants for analyzing the final microstructure, hardness, and austenite transformation temperatures in the cooling phase.

Variant Designation	Cooling Rate [°C/s]	Grain Size ECD ** [µm] Area 500 × 500 µm, Step 0.2 µm, Average Value/Standard Deviation
H5 C0.03	0.03	26.2/15.94
H100 C0.1	0.1	15.0/8.87
H1 C0.3	0.3	10.0/7.89
H10 C1	1	9.0/6.98
H50 C3	3	9.4/6.01
H50 C10	10	5.7/4.22
H0.1 C10	10	5.8/4.71
H50 C30	30	4.4/2.71
H0.5 C30	30	4.9/3.17
H250 C100	100	5.3/3.76
H250 C100 *	100 *	4.9/2.89
H50 C200	200	5.8/4.64
H50 C200 *	200 *	5.1/3.19
As rolled	-	4.1/2.34

* without delay, ** equivalent circle diameter.

## Data Availability

Data sharing is not applicable to this article.
